# An open-source video tracking system for mouse locomotor activity analysis

**DOI:** 10.1186/s13104-020-4916-6

**Published:** 2020-01-30

**Authors:** Chen Zhang, Haiwen Li, Renzhi Han

**Affiliations:** Department of Surgery, Davis Heart and Lung Research Institute, Biomedical Sciences Graduate Program, Biophysics Graduate Program, The Ohio State University Wexner Medical Center, Columbus, OH 43210 USA

**Keywords:** Video tracking, Locomotor activity, Neuromuscular diseases, Mice

## Abstract

**Objective:**

The ability to accurately and efficiently quantify mouse locomotor activity is essential for evaluating therapeutic efficacy and phenotyping genetically modified mice, in particular for the research of neuromuscular diseases. Our objective is to develop a program for video tracking of mice and locomotion analysis.

**Results:**

Here we describe a MATLAB program for video tracking of mice and locomotion analysis. The system is composed of a webcam, an open field, and a computer with MATLAB installed. Animal behavior is recorded by the webcam and the video is then analyzed for mouse position on each frame by a customized MATLAB code. The system has been tested for analyzing two or more mice simultaneously placed in individual chambers. The accumulative moving distance, velocity and thigmotaxis (percentage of time spending in the outer peripheral of the arena, which is commonly used as an index of anxiety) within a test period can be readily obtained. This system can be easily implemented in any laboratory as an in vivo locomotion assay to assess the neuromuscular abnormality of genetically modified animals and the impact of therapeutic interventions.

## Introduction

Measurement of position and locomotion are central to the study of animal behavior, such as novelty exploration, anxiety, spatial orientation and learning/memory. The Open Field Test (OFT) was first described by Hall and Ballachey in 1932 [1] and is still one of the most commonly used assays for monitoring exploratory behavior and locomotor activity in laboratory animals. Moreover, the OFT has been used to assess neuromuscular effects across a variety of experimental paradigms, such as stroke, psychotic effects of drug, stress/anxiety, aging, gender, circadian cycling, neuromuscular diseases of genetic origin, and environmental factors.

Despite its worldwide popularity among the scientific community, the vast majority of research laboratories still rely on commercial systems for recording and analyzing the OFT data. There are two major types of commercial OFT systems, photocell-based [2] and video-based [3, 4]. Examples of photocell-based commercial systems include Opto-Varimex (Columbus Instruments, OH, USA) and IR Actimeter (PanLab), while ANY-maze (Stoelting Co, IL, USA) and Ethovision^®^ XT (Noldus, Wageningen, The Netherlands) are based on video tracking. These commercial systems are excellent in their recording and analytical capability, however, they are typically provided with specific hardware and software bundled and thus relatively expensive. Moreover, they offer little methodological transparency or flexibility.

Due to the low requirement on the hardware for video tracking as compared to photocell-based systems, it becomes more attractive for the research laboratories to build up their own customized video-tracking system for mouse behavior analysis. Several open source programs have been reported as alternatives to the commercial OFT systems [5–13]. These programs can all allow the determination of travel distance and time spent within a defined region-of-interest (ROI). However, their performance varies in terms of simultaneous multi-animal tracking capability, running speed, data output, and capacity for batch analysis.

Here we describe MouseActivity—an open source MATLAB script that offers rapid analysis of locomotion parameters, such as distance travelled, mean speed, thigmotaxis, and stationary fraction. Using a mouse model of Inflammatory Bowel Disease (IBD) and a mouse model of Duchenne muscular dystrophy (DMD), we show that MouseActivity can quantitatively discern differences in movement across an Open Field. The script is freely available at https://github.com/HanLab-OSU/MouseActivity for the scientific community so that it can be tailored and customized for the specific settings and purposes in different laboratories. The script has been tested in MATLAB R2018b under both Windows7 and macOS High Sierra. An executable installer has also been compiled for the macOS system.

## Main text

### Methods

#### Mice

Mice (C57BL/6J and B6Ros.Cg-*Dmd*^*mdx*−*4Cv*^/J) were maintained at The Ohio State University Laboratory Animal Resources in accordance with animal use guidelines. After the final video recording, mice were sacrificed by CO_2_ inhalation. All animal studies were authorized by the Animal Care, Use, and Review Committee of the Ohio State University.

#### Establishment of an inflammatory bowel disease (IBD) mouse model

Drinking water containing 3% (w/v) dextran sulfate sodium (DSS, 36,000–50,000 Da, MP Biomedicals, Solon, OH) was given to male C57BL/6J mice at 20 weeks of age for 8 days [14]. The mice were video-recorded before and on day 8 after the initiation of DSS treatment. They were also weighed every day to monitor the body mass change.

#### Open field setup and video recording

The open fields are composed of one, two or four 12″ × 12″ × 12″ transparent acrylic chambers aligned horizontally or vertically, with each chamber housing a single mouse to be analyzed (see Additional file 1). We have tested our system for analyzing one mouse or two mice simultaneously, but it can be easily modified to analyze more mice simultaneously. Regular mouse bedding can also be included if preferred. A Microsoft Surface Pro 4 Tablet with its built-in rear camera was used to record the mouse videos for behavior analysis. The videos were acquired in the MP4 format with a resolution of 1920 × 1080 pixels at 30 frames per second.

#### Video processing and data analysis

The video files are processed by a MATLAB script (version R2018b), running on a PC with Microsoft Windows 7 or an Apple MacBook Air computer with macOS High Sierra (version 10.13.6). The MouseActivity code was designed to detect black mice such as C57BL/6J and *mdx*^*4cv*^ in a light arena, but it can be easily modified to do the opposite (e.g. light-colored animals in a dark arena). The MouseActivity processes the movie files frame-by-frame continuously or in steps, detects the mice in each frame, determines the position of the mice, and save the data. The MouseActivity source code is freely available at https://github.com/HanLab-OSU/MouseActivity.

#### Statistical analyses

Statistical analyses were performed using GraphPad Prism^®^ 5.02 (GraphPad Software, Inc.). Results are expressed as Mean ± S.E.M. The number of independent experiments and the statistical test employed are indicated in the respective figure legends. A *p* value less than 0.05 was considered to be statistically significant.

### Results

#### Workflow of the MouseActivity

The core of the program (Additional file 2) works by first inverting the grayscale image of each video frame, converting it to binary image based on a user-defined threshold (using the ‘im2bw’ function), removing all connected objects that have fewer than designated pixels (the background noise other than the mouse) from the binary image (using the ‘bwareaopen’ function), and measuring the position/area of the mouse in the binary image (using the ‘regionprops’ function). After finding the position of the animal, the program proceeds to the next video frame. It can also be set to skip video frames using a parameter called “steps”, which has a default value of 3 (meaning every 3rd frame will be analyzed). By identifying the position of the mice on each frame, the software can then compute the movement properties such as traveling distance, average speed and thigmotaxis, and output the data into Microsoft Excel format for image plotting and statistical analysis.

**Fig. 1:**
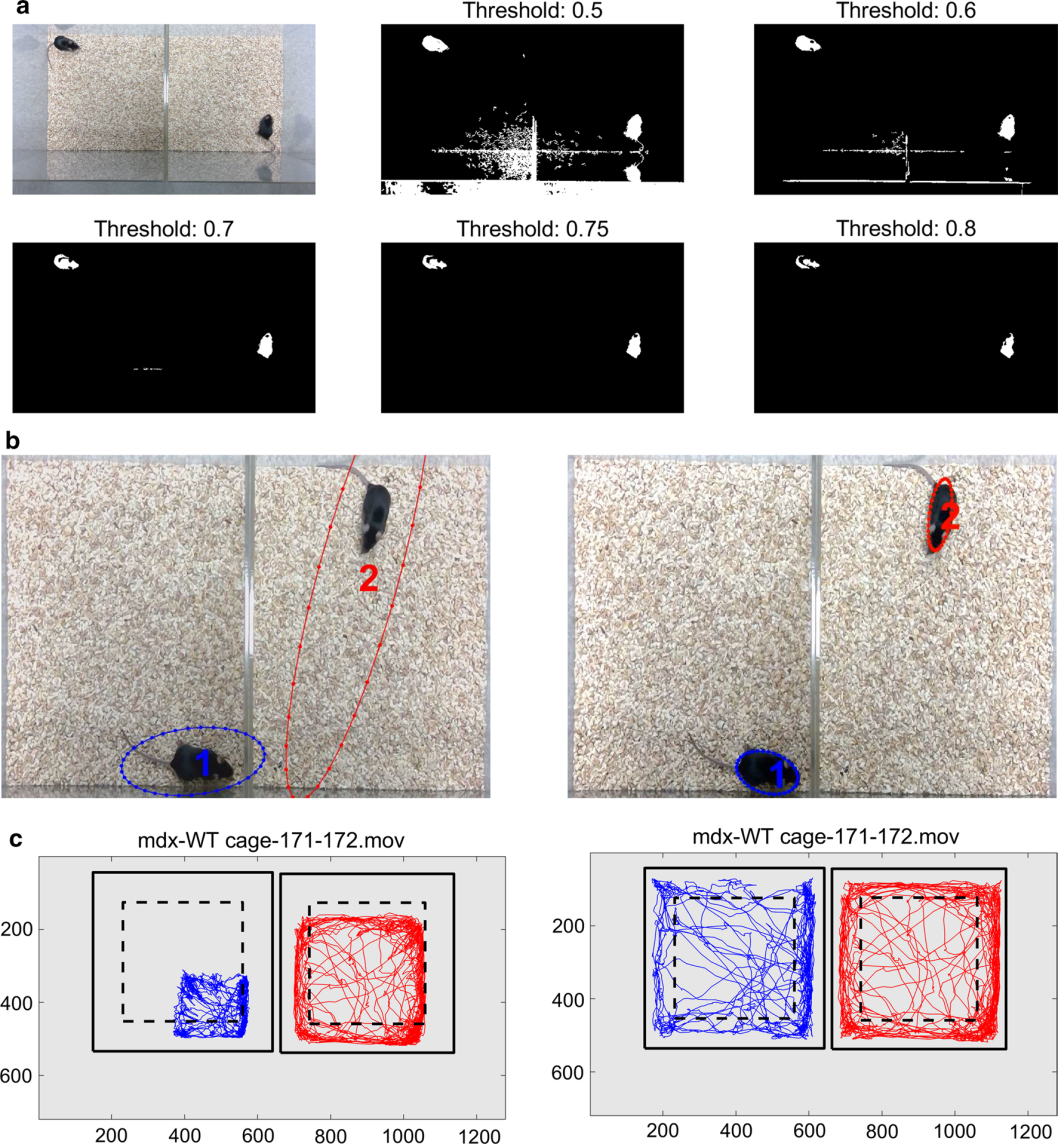
The impact of different thresholding level on identification of the mice in the video frames. **a** Increase of threshold from 0.5 to 0.8 gradually improved the signal/noise ratio of the mice images. **b** The identification of mouse in a video frame as marked by the color ovals at the threshold of 0.5 (left) or 0.75 (right). **c** The mouse trajectory plot of the mice with the threshold of 0.5 (left) or 0.75 (right)

Once started, the program will ask for the selection of a movie file (a sample video file is provided in Additional file 3 for testing) to analyze. It will then pop up a figure showing one of the frames with different thresholding levels (Fig. 1). This gives the user an idea about what threshold may be ideal for the image processing. The threshold setting is critical in image segmentation. As shown in Fig. 1a, increasing the threshold from 0.5 to 0.8 gradually improved the discernibility of the mice from the background noises. To illustrate the importance of setting a proper threshold value on tracking of mouse, we compared the tracking results of a test movie file with a threshold of 0.5 and 0.75. At the threshold of 0.5, both mice were labeled with much larger ovals than the mouse bodies (Fig. 1b, left) indicating that the incorrect identification of the mouse positions. In contrast, at the threshold of 0.75, the color ovals marked each mouse precisely (Fig. 1b, right). The trajectory of each mouse was obviously shifted when the threshold was set at 0.5 (Fig. 1c, left) as compared to 0.75 (Fig. 1c, right).

After clicking the image with the best threshold, the program will ask the user to enter several other parameters including the minimal pixel size of the objects (mice), the number of animals in the movie, the start frame and last frame to be analyzed, and the frame steps to be analyzed (e.g. analyze every “x” frame). The default values for all these parameters can be over-written here by manual input. The minimal pixel size of the mouse is also important for correct identification of the mouse objects. These parameters need to be adjusted according to the video specification.

After completion of the tracking process in all frames, the program will save the tracking data (e.g. the position coordinates and area of each mouse) into a MATLAB file (Additional file 4), and start to analyze the data. This will result in several outputs including a summary figure with mouse trajectory and travel distance (Additional file 5), a figure with the distribution of each mouse area, eccentricity and major axis length (e.g. the mouse body length) (Additional file 6), and an Excel file with the mouse position, travel distance and thigmotaxis (Additional file 7). When both tracking and analysis have been completed, it will ask the user if they want to check the tracking results. If yes is checked, it will pop up a dynamic figure showing each analyzed frame with the mouse labeled by a color oval and number. After completing this process, it will save the tracking movie (Additional file 8) for examination of the tracking accuracy.

#### Evaluation of mice with IBD

**Fig. 2:**
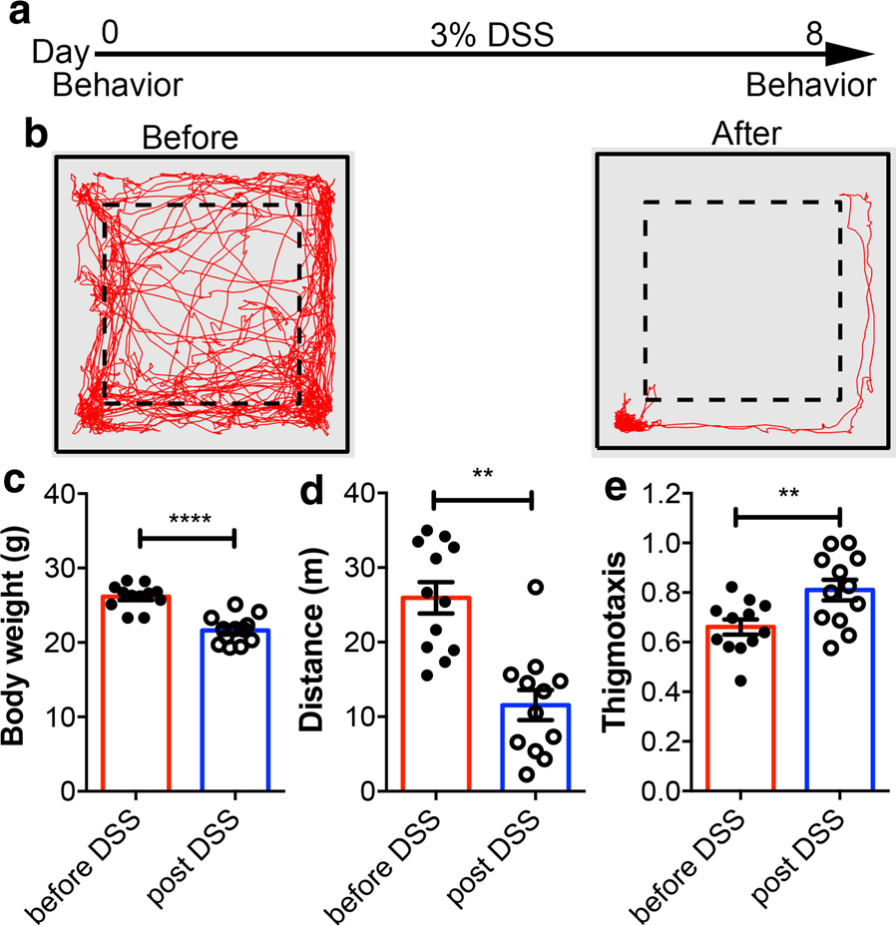
Evaluation of IBD mice using MouseActivity. **a** Diagram showing the time points of IBD induction and the video recording. **b** A representative trajectory plot of a WT mouse before and after 8-days of IBD induction. Body mass (**c**), travel distance (**d**) and thigmotaxis (**e**) of the mice before and after IBD induction. Paired t-test was used to determine the statistical significance. **p < 0.01; ****p < 0.0001

Before the DSS treatment (Fig. 2a), the mean body weight of mice was 26.16 ± 0.47 g (n = 12). During the IBD induction, the mice showed a steady weight loss (Fig. 2c). On the 8th day of DSS treatment, the mean body weight of mice was reduced by ~ 17% to 21.63 ± 0.54 g. We then used the MouseActivity software to analyze the mouse activity changes before and after the induction of IBD in an Open Field recorded in 10-min videos. The mouse trajectory (Fig. 2b) demonstrated that the mice moved much less across the Open Field after the DSS treatment than before the DSS treatment. On average, the mice travelled 26.0 ± 2.1 m in 10 min before the DSS treatment, whereas the total travel distance was significantly decreased to 11.6 ± 2.0 m within 10 min after the DSS treatment (Fig. 2d). IBD induction may also affect the anxiety of the mice. Indeed, we observed that the mice after IBD induction tended to stay longer at the peripheral of the Open Field as reflected by an increased thigmotaxis value (Fig. 2e).

#### Evaluation of mice with Duchenne muscular dystrophy

**Fig. 3:**
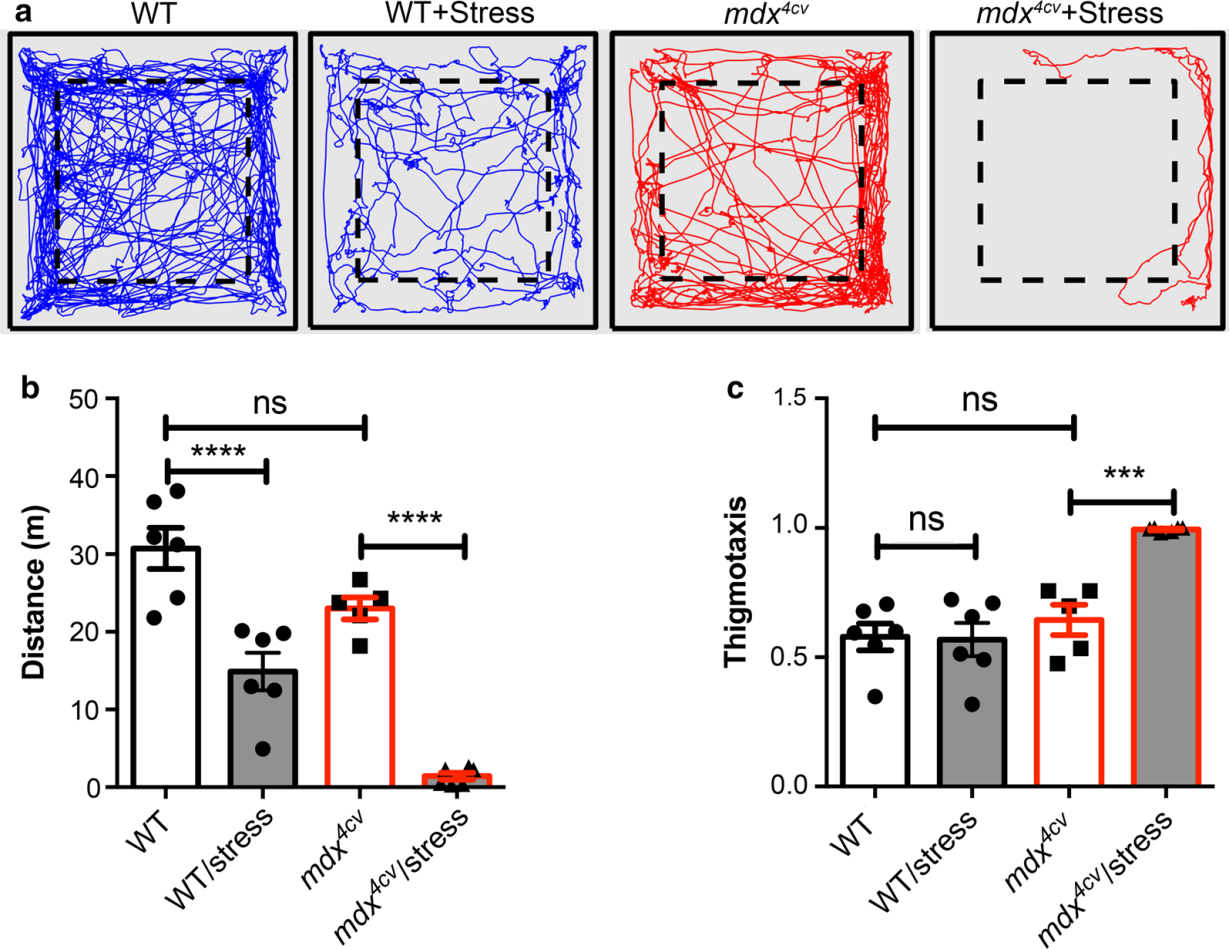
Evaluation of *mdx*^*4cv*^ mice using MouseActivity. **a** A representative trajectory plot of a WT and *mdx*^*4cv*^ mouse without or with grabbing/release stress. The travel distance (**b**) and thigmotaxis (**c**) of the mice without or with grabbing/release stress. One-way ANOVA with Bonferroni’s multiple comparisons test was used to determine the statistical significance. ****p < 0.0001; ***p < 0.001; *ns* not significant

Loss of dystrophin in Duchenne muscular dystrophy (DMD) leads to muscle degeneration and regeneration, and impairs the contractile function of muscles [15]. To examine the physical activity of *mdx*^*4cv*^ mice, a mouse model of DMD [16], we recorded 10-min videos of wild-type (WT, C57BL/6J) and *mdx*^*4cv*^ mice at the age of 20 weeks in the Open Field and analyzed the videos using MouseActivity. A representative trajectory was shown in Fig. 3a for each strain. Under the basal condition, the *mdx*^*4cv*^ mice showed a trend to travel less than WT mice within 10 min (30.8 ± 2.7 vs. 23.0 ± 1.4 meters, not significant) (Fig. 3b). There was no significant difference in the thigmotaxis between WT and *mdx*^*4cv*^ mice (Fig. 3c). To examine how the mice respond to stress, we grabbed the mice by the tail and neck skin, and then released them for free, and this was repeated for 5 times before placing them in the Open Field for video recording. Interestingly, this mild stress significantly reduced the average travel distance of WT mice by about half (Fig. 3a), and more dramatic reduction (~ 93.8%) was observed in the *mdx*^*4cv*^ mice, which essentially stayed quiet in the corner of the Open Field during the entire observation time window (Fig. 3a–c), indicating that the *mdx*^*4cv*^ mice had an increased anxiety after stress.

## Limitations

In this study, we developed a MATLAB program, MouseActivity, as an alternative open-source video tracking system for mouse locomotor activity analysis. This system does not require sophisticated hardware, and can be run on an average personal computer with MATLAB installed. However, the users will be required to build their own Open Field and movie recorder, which are straightforward and can be as simple as placing a rectangular chamber with an overhead webcam (most of Laptop computer has a high-quality webcam pre-installed). The script is currently written to analyze only rectangular Open Field, but can be modified easily to analyze Open Field of other shapes. Another limitation of MouseActivity is that it could not analyze two or more mice placed in a single Open Field, and is incapable of distinguishing the animal’s head from its tail.

## Supplementary information

**Additional file 1.** A sample Open Field composed of two 12″ × 12″ × 12″ transparent acrylic chambers, with a mouse in each chamber.

**Additional file 2.** Flowchart of overall execution of the MouseActivity program.

**Additional file 3.** A sample 10-min video file of two mice in their Open Field.

**Additional file 4.** A sample MATLAB data output of the MouseActivity program after completing the tracking process of a movie, which include the position coordinates and area of each mouse.

**Additional file 5.** A sample summary output after analyzing a video of two mice using MouseActivity. (**A**) Mouse trajectory showing two mice in their own Open Field. The solid black lines indicate the border of the Open Field; the dash line boxes outline the center area of the Open Field; the space between the solid lines and the dash lines were designated as the peripheral area of the Open Field. (**B**) The accumulative travel distance of individual mice in 10-min of recording.

**Additional file 6.** A sample figure output of the MouseActivity program showing the distribution of each mouse area, eccentricity and major axis length (e.g. the mouse body length).

**Additional file 7.** A sample Excel file output of the MouseActivity program with the data of the mouse position, travel distance and thigmotaxis.

**Additional file 8.** A sample tracking movie saved by the MouseActivity program with each mouse labelled by a color oval (e.g. red or blue) and number (e.g. mouse 1 or 2).

## Data Availability

All analyzed data during this study are included in this manuscript and its supplementary information files, and the original datasets during the current study are available from the corresponding author on reasonable request. The software codes are publicly available at: https://github.com/HanLab-OSU/MouseActivity.

## Abbreviations

DMD: Duchenne muscular dystrophy
DSS: Dextran sulfate sodium
IBD: Inflammatory bowel disease
OFT: Open field test
ROI: Region-of-interest
WT: Wild-type
