# Supplementary figures and images for: An open-source video tracking system for mouse locomotor activity analysis
Source: BMC Res Notes. 2020 Jan 30;13:48. doi: 10.1186/s13104-020-4916-6 (PMC6990588; doi:10.1186/s13104-020-4916-6)

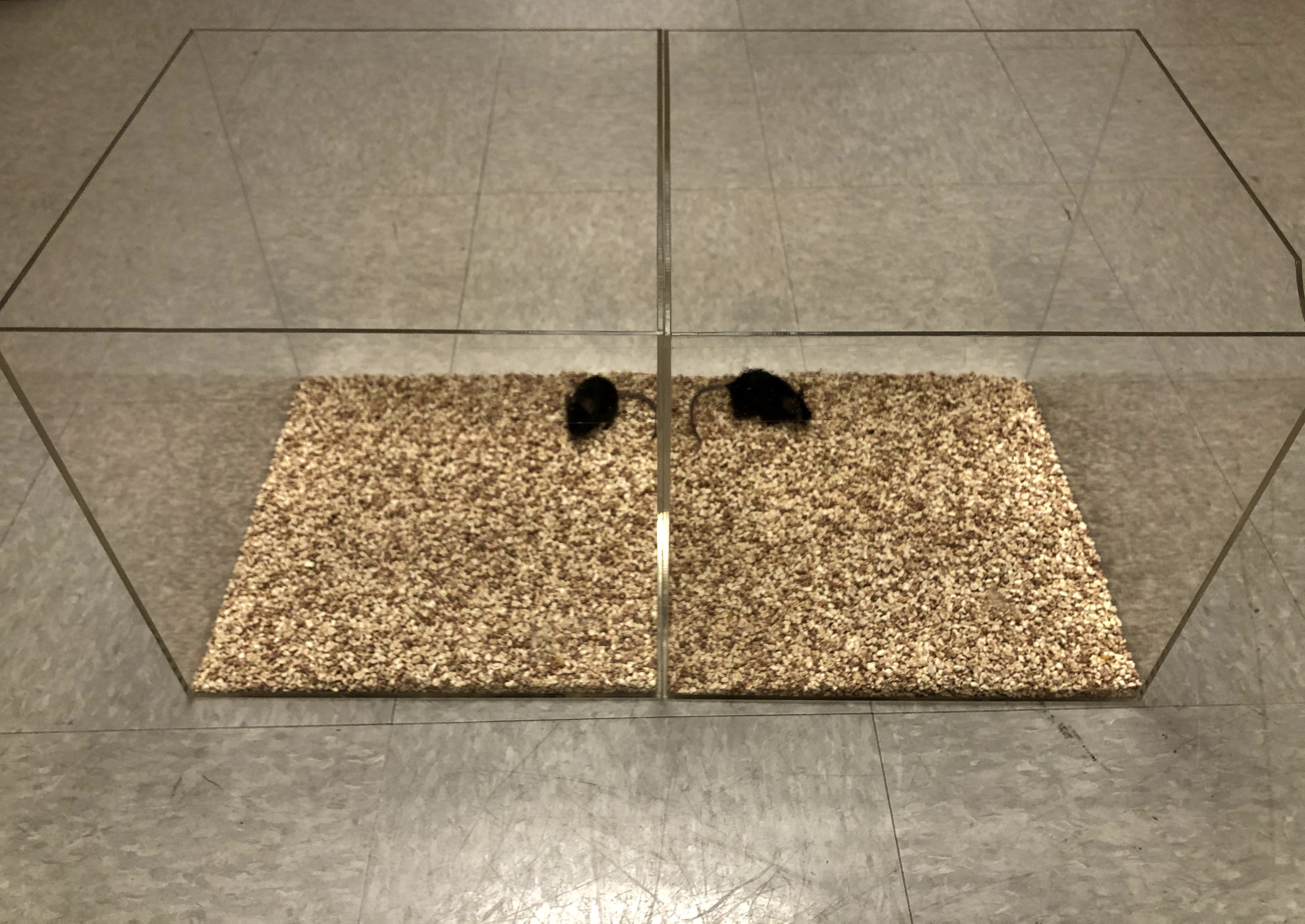

Supplement: Supplementary file 1 — Additional file 1. A sample Open Field composed of two 12″ × 12″ × 12″ transparent acrylic chambers, with a mouse in each chamber. [file 13104_2020_4916_MOESM1_ESM.jpg]

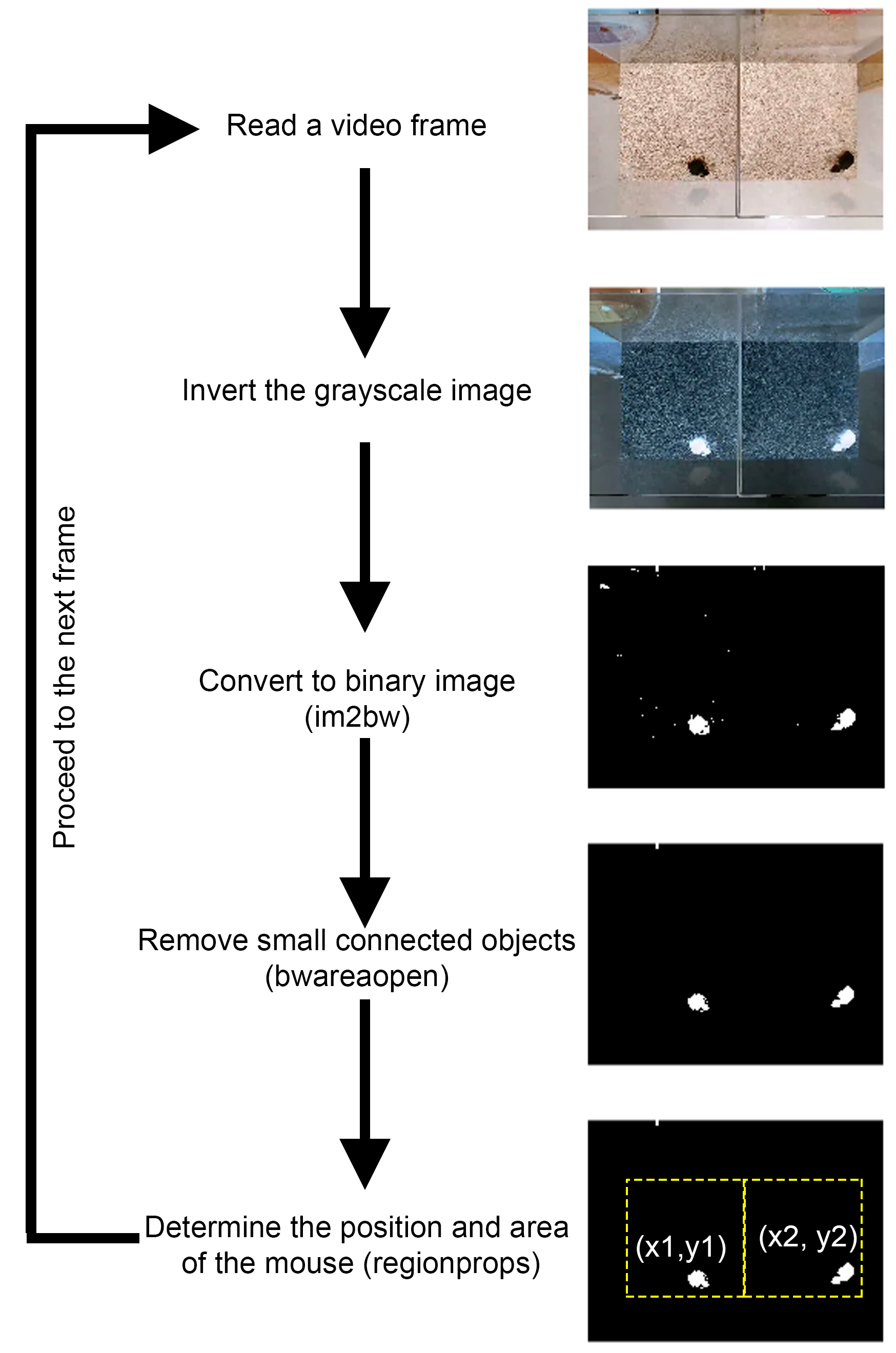

Supplement: Supplementary file 2 — Additional file 2. Flowchart of overall execution of the MouseActivity program. [file 13104_2020_4916_MOESM2_ESM.tif]

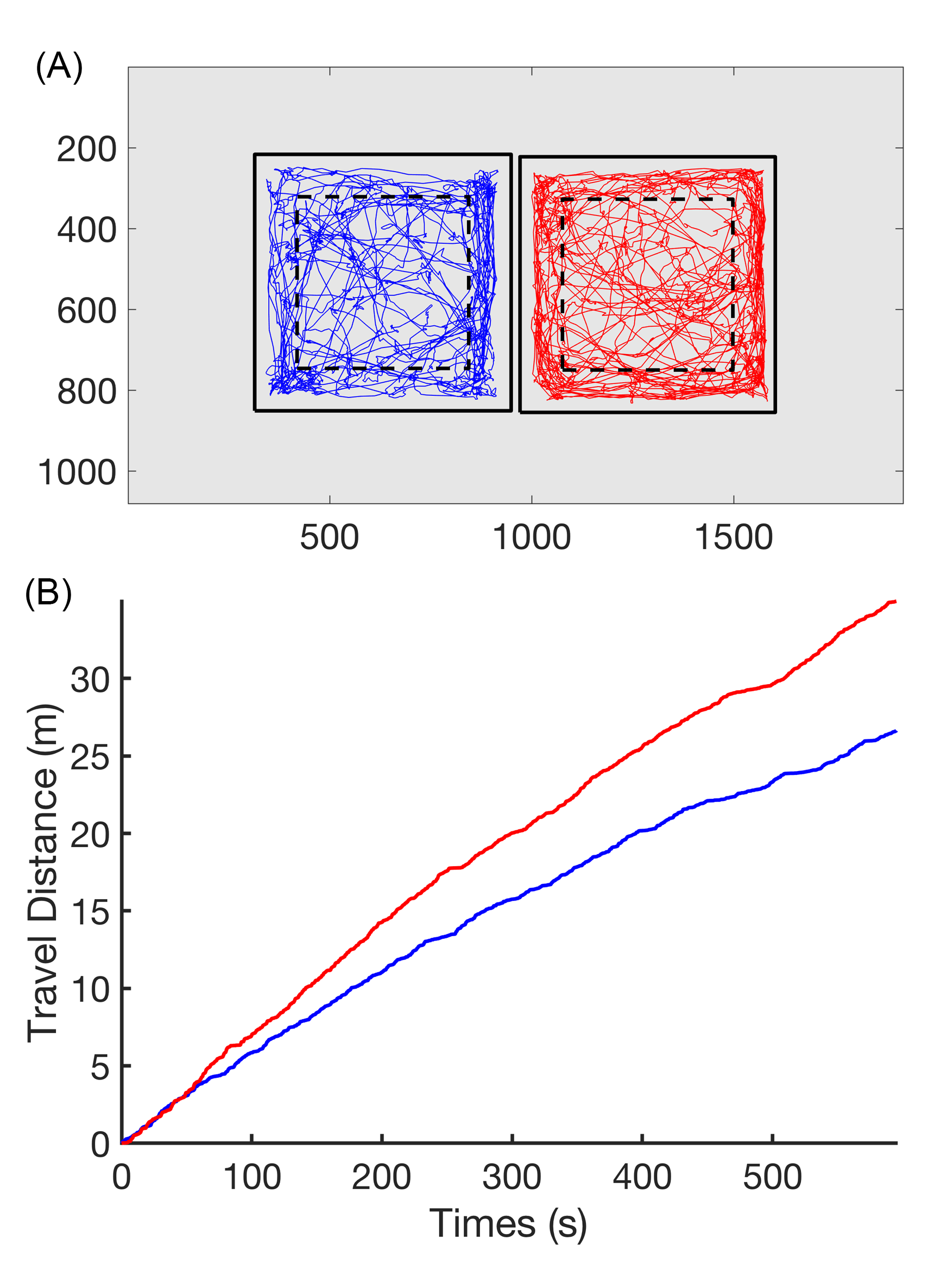

Supplement: Supplementary file 5 — Additional file 5. A sample summary output after analyzing a video of two mice using MouseActivity. (A) Mouse trajectory showing two mice in their own Open Field. The solid black lines indicate the border of the Open Field; the dash line boxes outline the center area of the Open Field; the space between the solid lines and the dash lines were designated as the peripheral area of the Open Field. (B) The accumulative travel distance of individual mice in 10-min of recording. [file 13104_2020_4916_MOESM5_ESM.tif]

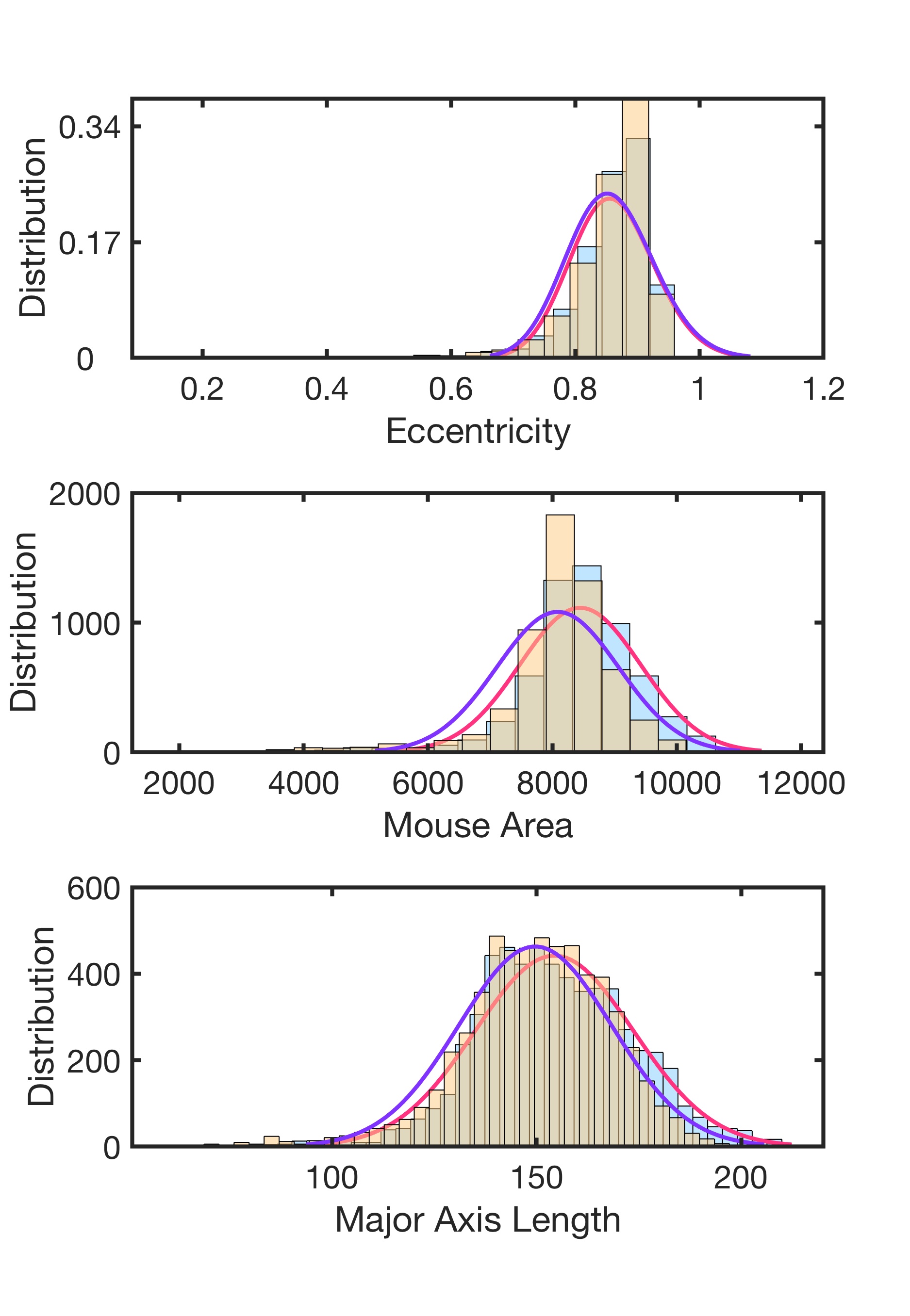

Supplement: Supplementary file 6 — Additional file 6. A sample figure output of the MouseActivity program showing the distribution of each mouse area, eccentricity and major axis length (e.g. the mouse body length). [file 13104_2020_4916_MOESM6_ESM.tif]
